# Effects of step frequency during running on the magnitude and symmetry of ground reaction forces in individuals with a transfemoral amputation

**DOI:** 10.1186/s12984-022-01012-8

**Published:** 2022-03-23

**Authors:** Toshiki Kobayashi, Mark W. P. Koh, Mingyu Hu, Hiroto Murata, Genki Hisano, Daisuke Ichimura, Hiroaki Hobara

**Affiliations:** 1 Department of Biomedical Engineering, Faculty of Engineering, The Hong Kong Polytechnic University, Hong Kong, China; 2 Artificial Intelligence Research Center, National Institute of Advanced Industrial Science and Technology (AIST), Waterfront 3F, 2-3-26, Aomi, Koto-ku, Tokyo, 135-0064 Japan; 3 Department of Mechanical Engineering, Tokyo University of Science, Chiba, Japan; 4 Department of Systems and Control Engineering, Tokyo Institute of Technology, Tokyo, Japan; 5 Research Fellow of Japan Society for the Promotion of Science (JSPS), Tokyo, Japan

**Keywords:** Amputee, Running-specific prosthesis, Running, Gait, Ground reaction forces, Kinetics

## Abstract

**Background:**

Individuals with unilateral transfemoral amputation are prone to developing health conditions such as knee osteoarthritis, caused by additional loading on the intact limb. Such individuals who can run again may be at higher risk due to higher ground reaction forces (GRFs) as well as asymmetric gait patterns. The two aims of this study were to investigate manipulating step frequency as a method to reduce GRFs and its effect on asymmetric gait patterns in individuals with unilateral transfemoral amputation while running.

**Methods:**

This is a cross-sectional study. Nine experienced track and field athletes with unilateral transfemoral amputation were recruited for this study. After calculation of each participant’s preferred step frequency, each individual ran on an instrumented treadmill for 20 s at nine different metronome frequencies ranging from − 20% to + 20% of the preferred frequency in increments of 5% with the help of a metronome. From the data collected, spatiotemporal parameters, three components of peak GRFs, and the components of GRF impulses were computed. The asymmetry ratio of all parameters was also calculated. Statistical analyses of all data were conducted with appropriate tools based on normality analysis to investigate the main effects of step frequency. For parameters with significant main effects, linear regression analyses were further conducted for each limb.

**Results:**

Significant main effects of step frequency were found in multiple parameters (P < 0.01). Both peak GRF and GRF impulse parameters that demonstrated significant main effects tended towards decreasing magnitude with increasing step frequency. Peak vertical GRF in particular demonstrated the most symmetric values between the limbs from − 5% to 0% metronome frequency. All parameters that demonstrated significant effects in asymmetry ratio became more asymmetric with increasing step frequency.

**Conclusions:**

For runners with a unilateral transfemoral amputation, increasing step frequency is a viable method to decrease the magnitude of GRFs. However, with the increase of step frequency, further asymmetry in gait is observed. The relationships between step frequency, GRFs, and the asymmetry ratio in gait may provide insight into the training of runners with unilateral transfemoral amputation for the prevention of injury.

**Supplementary Information:**

The online version contains supplementary material available at 10.1186/s12984-022-01012-8.

## Background

Running has been a key method of locomotion for mankind. Although it has become a common competitive sport for some amputee athletes who can run again, many individuals with lower limb amputation still find it challenging. According to a previous study, sixty percent of individuals who suffered combat-related amputations reported a complete inability to run one block with a prosthesis [1]. It has been well documented that individuals with amputation develop a more asymmetric gait pattern, especially while running. The asymmetric gait pattern causes increased ground reaction forces (GRFs) on the intact limb, leading to a higher probability to develop health conditions such as knee osteoarthritis and musculoskeletal injuries [2–4]. The increased GRFs affect the loading rates on the joints which have been associated with the expanded occurrences of these health conditions [5]. Both lateral and medial forces have been correlated with elevated probabilities of developing knee osteoarthritis, although the medial condition is more common than the lateral [6, 7]. Therefore, reduction in GRFs on the intact limb and more symmetric gait pattern may prevent possible future injuries or disease in individuals with lower limb amputation.

Asymmetric gait can be evaluated by analyzing gait cycle, step mechanics, range of motion, and the composing values of GRFs [8]. Individuals with lower limb amputation have an obvious difference in symmetry of gait when compared to able-bodied individuals [9, 10], and gait asymmetry comes with significant increases in load on the intact limb [11]. The prosthetic limb is unable to duplicate the control and mechanism of the intact limb [12] making asymmetric gait unavoidable. The individuals with unilateral transfemoral amputation are likely, subconsciously or not, to rely more on the intact limb. With the increase of walking velocity or into running, the asymmetry tends to be amplified [5, 13], causing increased loading stress on the intact limb [14, 15]. The increased loading stress can be affected by an increase of factors such as vertical GRFs [16, 17], mediolateral GRFs [6], breaking and propulsive impulses [18], as well as spatiotemporal parameters such as stance time [5]. Anteroposterior GRFs have also been hypothesized to be one of the potential factors in injuries related to running [19].

For an able-bodied individual, increasing cadence or step frequency during gait can decrease GRFs on the lower limb joints [20–24]. Increasing step frequency can also reduce the heart rate of runners as well as oxygen costs when the speed remains constant [25], which can prove beneficial to individuals with transfemoral amputation as they expend significantly more energy compared to those who are able-bodied during gait [26–28]. However, there has been a paucity of studies on the effects of step frequency on GRFs in individuals with amputation [29, 30]. Step frequency manipulation is one of the fundamental locomotor strategies, and there should be an optimum step frequency to minimize GRFs on the intact limb. It has been reported that able-bodied individuals can be trained to vary step frequency at a given running speed resulting in changing GRFs [31, 32]. Thus, retraining of step frequency in individuals with amputation should also be viable [29, 30].

Therefore, the first aim of the present study was to investigate the effects of step frequency on the magnitude of GRFs in the intact and prosthetic limbs of individuals with transfemoral amputation. The second aim was to investigate the effect of step frequency on the symmetry of GRFs and spatiotemporal parameters between intact and prosthetic limbs. The first hypothesis of this study was that increasing step frequency at a given speed will lower the magnitude of GRFs on the intact and prosthetic limbs. The second hypothesis was that the asymmetry of GRFs between the intact and prosthetic limbs will be minimized at the preferred step frequency, although individuals with amputation still run with an asymmetric pattern and loading on the intact limb may not be the minimum.

## Methods

### Participants

This cross-sectional study shared a participant pool with a previous study [30]. Nine individuals with transfemoral amputation with experience in the long jump or 100-m sprint were recruited. They also were members of track and field teams and had been sprint training for more than 5 years. The mean of their best recorded times for the 100-m spring in the past year was 17.59 ± 2.15 s. Their demographic characteristics are summarized in Table 1. Four participants wore the 1E90 Sprinter (categories 2 to 4, Ottobock, Duderstadt, Germany) and five participants wore the 1E91 Runner (categories 2 to 5, Ottobock, Duderstadt, Germany). All participants attached rubber soles to their running-specific prostheses. The Institutional Review Board (Environmental and Safety Headquarters, Safety Management Division, National Institute of Advanced Industrial Science and Technology) ethically approved the study, and we conducted it following the guidelines given in the Declaration of Helsinki (1983). Informed written consent was obtained from all participants (or guardians, in participant’s 8 situation) prior to the experiment.

**Table 1:** Demographic characteristics

Subject	Preferred *f*_step_ (Hz)	Trial speed (m/s)	Age (years)	Sex	Total mass (kg)	Height (m)	Amputated limb	Time since amputation (years)	Prosthetic knee	RSP model
1	2.53	2.11	36	M	59.8	1.61	Right	17.9	3S80	Runner 1E91 (cat.3)
2	2.65	2.00	31	M	59.7	1.65	Right	3.0	Cheetah Knee	Runner 1E91 (cat.2)
3	2.68	2.14	20	F	45.2	1.62	Left	3.5	3S80	Sprinter 1E90 (cat.3)
4	2.70	2.86	27	M	70.4	1.75	Right	6.2	3S80	Runner 1E91 (cat.4)
5	2.73	2.31	23	M	56.3	1.68	Left	20.0	3S80	Sprinter 1E90 (cat.3)
6	2.85	2.25	34	M	58.7	1.61	Left	21.0	3S80	Runner 1E91 (cat.5)
7	2.88	2.39	20	F	56.5	1.56	Right	5.7	3S80	Runner 1E91 (cat.3)
8	2.93	2.75	17	M	86.0	1.77	Right	3.5	3S80	Sprinter 1E90 (cat.4)
9	3.00	1.94	21	F	46.7	1.49	Right	10.0	3S80	Sprinter 1E90 (cat.2)
Mean	2.77	2.31	25.44		59.91	1.64		10.1		
(SD)	(0.14)	(0.30)	(6.45)		(11.6)	(0.08)		(7.1)		

*f*_step_: step frequency; RSP: running specific prosthesis; Total mass = Body mass + Prosthetic mass

### Experimental procedures

**Fig. 1:**
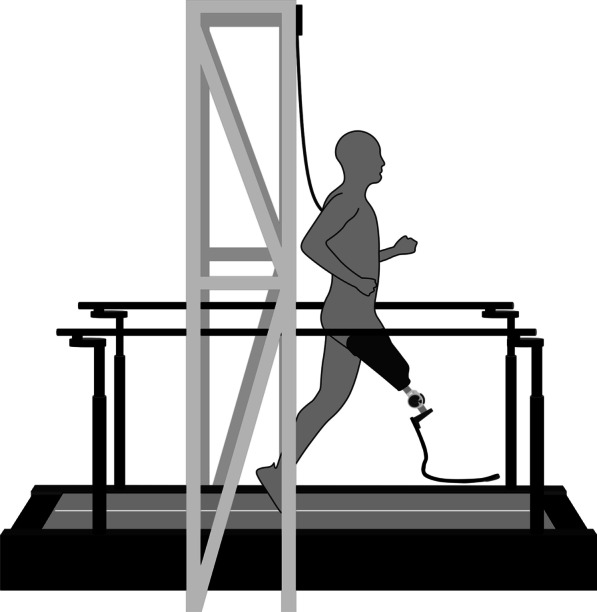
Experimental setup. Participants ran on a force-instrumented split-belt treadmill at nine metronome frequencies based on their preferred step frequency (− 20% to + 20% with increments of 5%) for 20 s at each frequency. The split-belt treadmill was embedded with two force plates. Two handrails and safety harness were fitted to avoid falling

An instrumented split-belt treadmill (FTMH-1244WA, Tec Gihan, Kyoto, Japan) was used in this study (Fig. 1). Participants were first instructed to walk and run for more than 5 min on the instrumented treadmill before the experiment in order to familiarize themselves with the equipment [33]. An estimation of each participant’s maximum speed was done by taking 100 m and dividing it by their personal best time for the 100-m sprint [34]. The treadmill was set for 40% of each individual's estimated maximum speed, and participants were instructed to run as normal for 20 s to determine each participant’s preferred step frequency (Table 1). In order to obtain a wide step frequency range, 40% of their estimated maximum speed was elected to be the running speed, as it was sufficiently low enough. The preferred step frequency was calculated by using 14 sequential steps taken during the middle of the trial. The inverse of the time from contact to the contralateral contact was used as the definition for the step frequency [30]. Preceding studies used a range of step frequency between − 30% and + 30% at a determined running speed [35–37]. Therefore, our participants were asked to run while matching a digital metronome beat at nine different step frequencies (metronome frequency): preferred (0%), four above (+ 5%, + 10%, + 15%, and + 20%), and four below (− 5%, − 10%, − 15%, and − 20%) the preferred step frequency. The participants were experienced runners using running-specific prostheses. They were given an adequate practice period and instructed to follow a target metronome beat as accurately as possible at each step frequency, before asking them to conduct one running trial for 20 s at each determined step frequency (with randomized order). They were also given rest periods between trials of 1 to 3 min to reduce the effects of fatigue. In this study, the mean values of the running speed (40% of participants’ maximum speed) and preferred step frequency were 2.31 ± 0.30 m/s and 2.77 ± 0.14 Hz, respectively (Table 1).

### Data collection

Two force platforms (TF-40120-CL and TF-40120-CR; Tec Gihan, Kyoto, Japan) integrated into the instrumented treadmill collected all components of GRF data (sampling at 1 kHz). The GRFs were further filtered by a fourth-order zero-lag low-pass Butterworth filter with a cut-off frequency at 25 Hz, according to the findings of a previous study [38]. Additionally, a 40-N threshold was applied for further vertical GRF analysis [39–42] to determine foot contact timing.

### Data analyses

In this study, data were calculated by averaging seven consecutive steps. As dependent variables, we determined spatiotemporal parameters (Additional file 1: Fig. S1 and Additional file 6: Material: equations for temporal parameters), such as contact time (the time that the applied force exceeded the threshold on the force plate) and aerial time (the time interval between the end of the contact period of one foot and the beginning of the contact period of the opposite foot) at each metronome frequency in each limb. We also determined swing time (time interval between foot-off to ipsilateral foot contact), step time (time interval between successive heel contacts), realized step frequency (the inverse of the time from touchdown to contralateral touchdown), and step length (the belt distance traveled between successive contact periods of opposite feet).

We also analyzed peak vertical GRF (vGRF), peak braking GRF, peak propulsive GRF, peak medial GRF, and peak lateral GRF across a range of metronome frequencies in both intact and prosthetic limbs. Additionally, the GRF impulses (GRIs) were calculated as time integrals of the GRFs over the stance phase. The medial, lateral, braking, and propulsive impulses were computed as time integrals of all negative and positive values of the mediolateral and anteroposterior GRFs. Net mediolateral and net anteroposterior impulses were calculated as the sum of the medial and lateral, and braking and propulsive impulses. Moreover, vertical GRI was calculated as time integrals of the vGRF. All GRF variables were normalized to the participant’s  total mass (body mass + prosthetic mass) and represented by a unit of body weight (BW). Thus, the GRIs relative to BW have a unit of BW-second (BWs).

Based on a previous study [43], the asymmetry ratio of spatiotemporal and peak GRFs and GRIs were calculated. For individuals with unilateral transfemoral amputation, the asymmetry ratio was calculated as the prosthetic limb’s data divided by the intact limb’s data.

$$Asymmetry \,ratio = \frac{Prosthetic\, limb's \,data}{{Intact \,limb's\, data}}$$

Therefore, 1.0 will denote perfect symmetry, and any deviation whether higher than 1.0 (prosthetic limb dominant) or lower than 1.0 (intact limb dominant) indicates asymmetry.

### Statistical analyses

We performed the Shapiro–Wilk test to confirm normality of data. If the data for each limb followed a normal distribution, we used one-way repeated measures ANOVA to investigate the main effects of step frequency in each limb. In contrast, if the data did not follow a normal distribution, the Friedman test was used. In both cases, we further used best-fit linear regression analyses when significant main effect was observed in each limb. In the analysis, coefficients of determination (*R*^2^), correlation coefficient and 95% confidence intervals (95% IC) were calculated. Treating the limb data as unpaired in both analyses represents a conservative approach and has been implemented in previous studies [44–46]. Statistical significance was set at 0.05 for ANOVA, Friedman test, and regression analyses. Statistically significant results at exact values less than 0.001 were represented as P < 0.001 by convention [47]. SPSS for Windows Version 26 (IBM, Armonk, NY, USA) was used for all the statistical analyses.

## Results

### Percent error of realized step frequency to metronome frequency

**Fig. 2:**
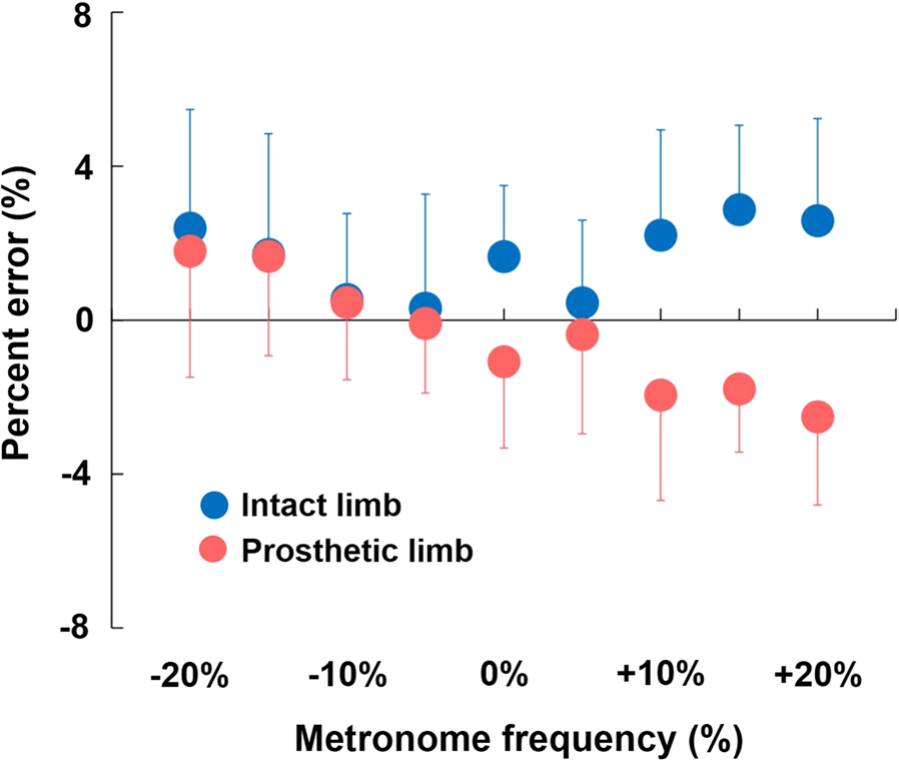
%Errors of realized step frequency to metronome frequency. Red and blue plots indicate prosthetic and intact limb, respectively

Percent error (%) of the realized step frequency from the metronome frequency is presented in Fig. 2. The mean error was within 4% of the metronome frequency. In the prosthetic limb, the error tended to be more negative at a higher metronome frequency and more positive at a lower metronome frequency. In the intact limb, the error tended to be more positive at either a higher or lower metronome frequency.

### Mean ground reaction forces

**Fig. 3:**
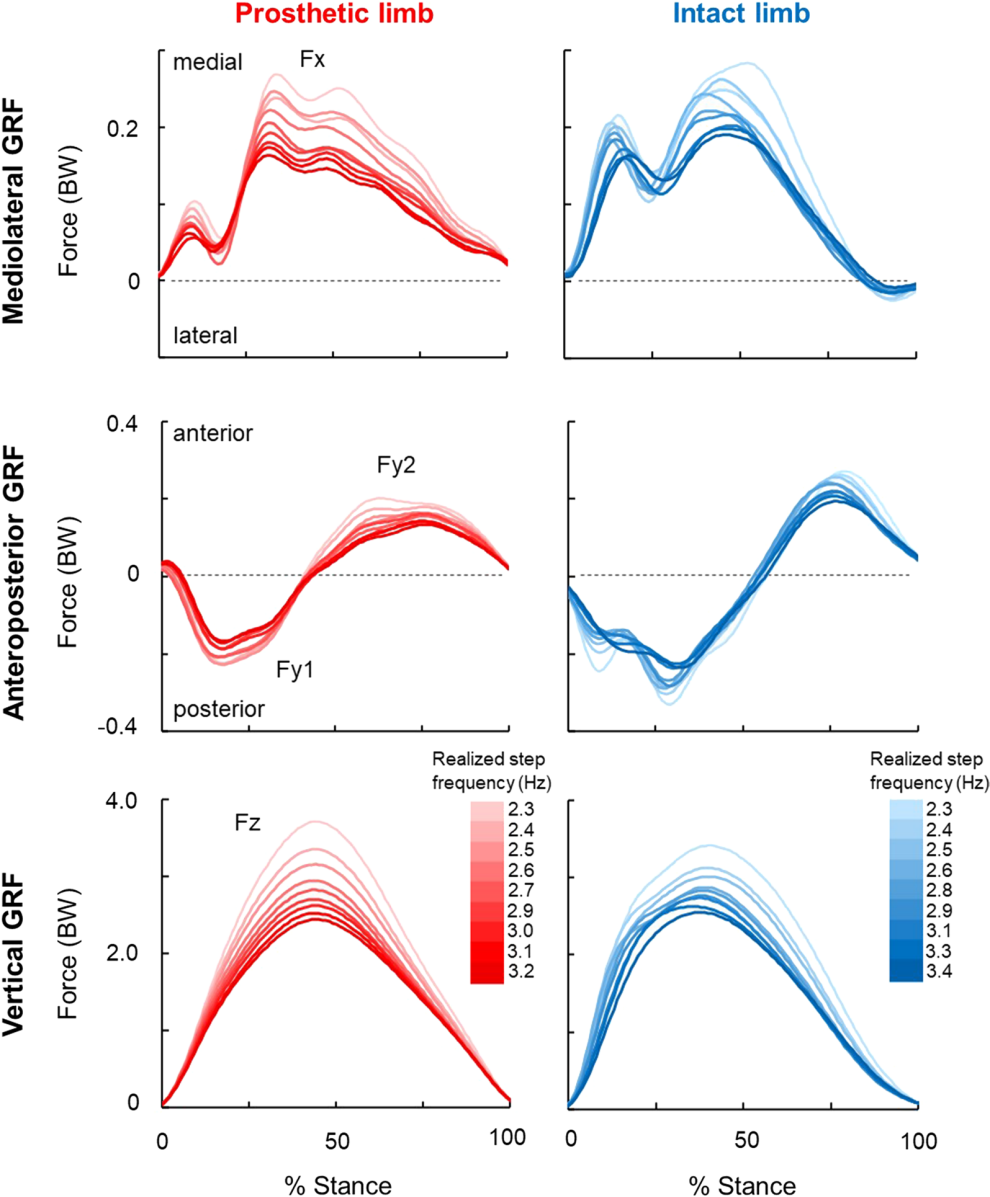
Mean ground reaction forces of both limbs across mean realized step frequencies at each metronome frequency. For the columns, left plot (red) is prosthetic limb, right plot (blue) is intact limb, respectively. For each row, top two plots are mediolateral GRF, middle two plots are anteroposterior GRF, and bottom two plots are vertical GRF. In each plot, the horizontal axis is normalized to stance duration from 0 to 100%, while the vertical axis is the forces (BW). The darkest and lightest colors represent highest and lowest realized step frequencies respectively. As the color lightens, step frequency decreases

Mean GRF (N = 9) across the mean realized step frequencies at each metronome frequency in mediolateral, anteroposterior, and vertical directions are presented in Fig. 3. For all GRF measurements (mediolateral, anteroposterior, and vertical), the magnitude at all stages of stance tends to decrease as step frequency increases. Overall, the prosthetic limb GRF measurements showed more regular and uniform changes across metronome frequency when compared to the intact limb as well.

### Spatiotemporal parameters

**Fig. 4:**
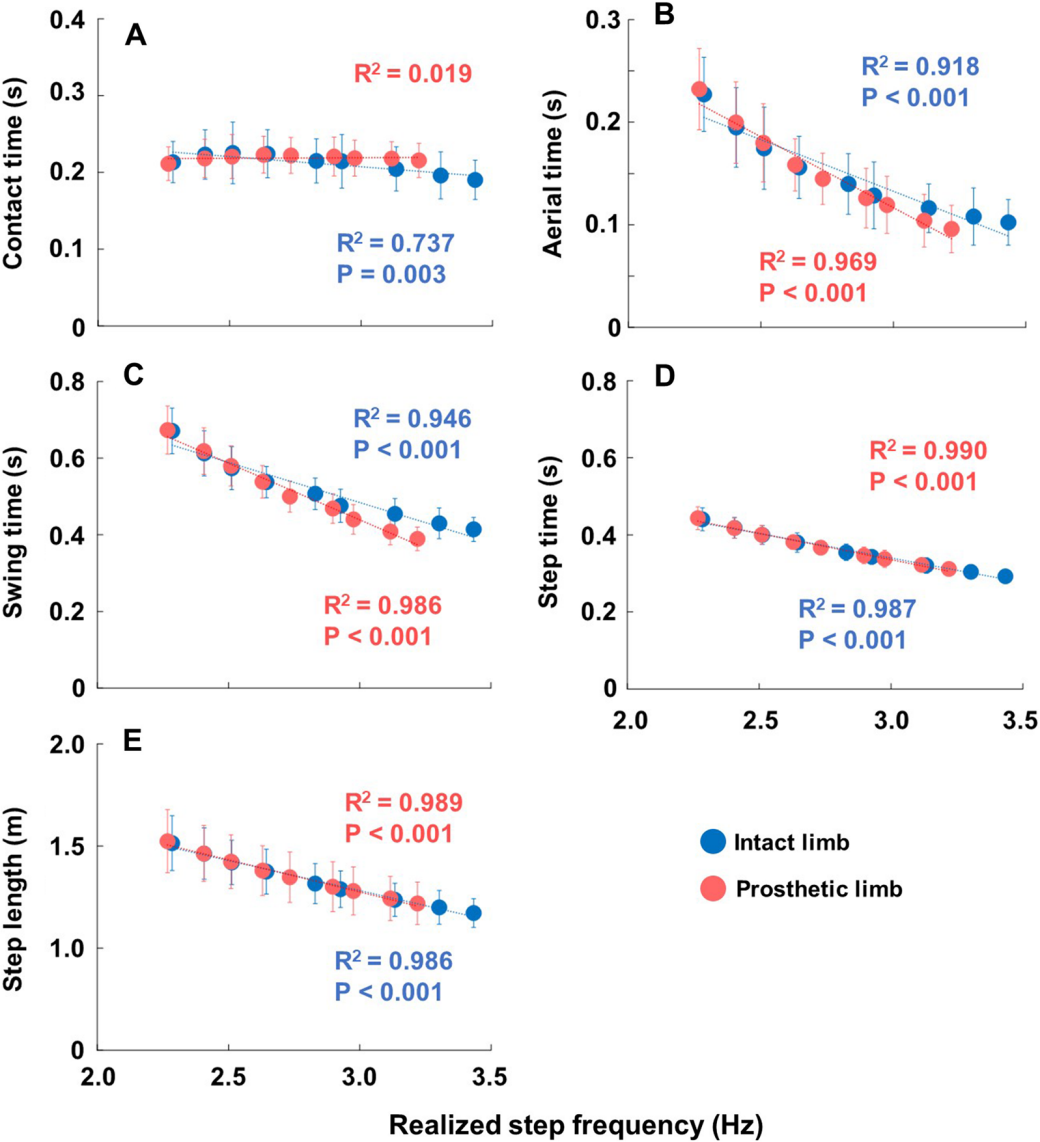
Mean spatiotemporal parameters and linear regressions of both limbs across mean realized step frequencies at each metronome frequency. In each plot, the horizontal axis represents realized step frequency. Each plot represents a different parameter. Parameters are represented in respective vertical axis as: contact time (s) (**A**), aerial time (s) (**B**), swing time (s) (**C**), step time (s) (**D**), and step length (m) (**E**). Each circle indicates the mean of all the participant’s data at each increment of metronome frequency from − 20% to + 20%, with blue indicating intact limb, and red indicating prosthetic limb. Significant main effects are indicated by filled circles. Relevant R^2^ and P values for the linear regressions are also displayed in matching color to the intact or prosthetic limb. P < 0.001 indicates P values which were at or smaller than 4 decimal places

Mean values and linear regressions with R^2^ values of each spatiotemporal parameter across the mean realized step frequencies at each metronome frequency are presented in Fig. 4. All the spatiotemporal parameters demonstrated significant main effects of step frequency, which are indicated by filled red (prosthetic limb) and blue (intact limb) circles in Fig. 4. Except for the contact time of the prosthetic limb (A), all parameters displayed statistical significances (P < 0.05) in linear regression analyses and exhibited a negative linear association with realized step frequency. The contact time (A) for the prosthetic limb was not statistically significant (R^2^ = 0.019). The contact time (A) in the intact limb (R^2^ = 0.737, P = 0.003) had a decreasing trend in relationship with increasing realized step frequency. Aerial time (B) for both the prosthetic limb (R^2^ = 0.969, P < 0.001) and the intact limb (R^2^ = 0.918, P < 0.001) exhibited a visible curved decrease with increasing realized step frequency, although still significant with linear association. Swing time (C) (prosthetic limb R^2^ = 0.986, P < 0.001; intact limb R^2^ = 0.946, P < 0.001), step time (D) (prosthetic limb R^2^ = 0.990, P < 0.001; intact limb R^2^ = 0.987, P < 0.001), and step length (E) (prosthetic limb R^2^ = 0.989, P < 0.001; intact limb R^2^ = 0.986, P < 0.001), all displayed nearly complete linear relationships with realized step frequency and decreased with increasing realized step frequency. Correlation coefficients, mean (SD), median (IQR), and 95% confidence intervals (95% CI) of the spatiotemporal parameters are summarized in Additional file 2: Table S1.

### Ground reaction force parameters

**Fig. 5:**
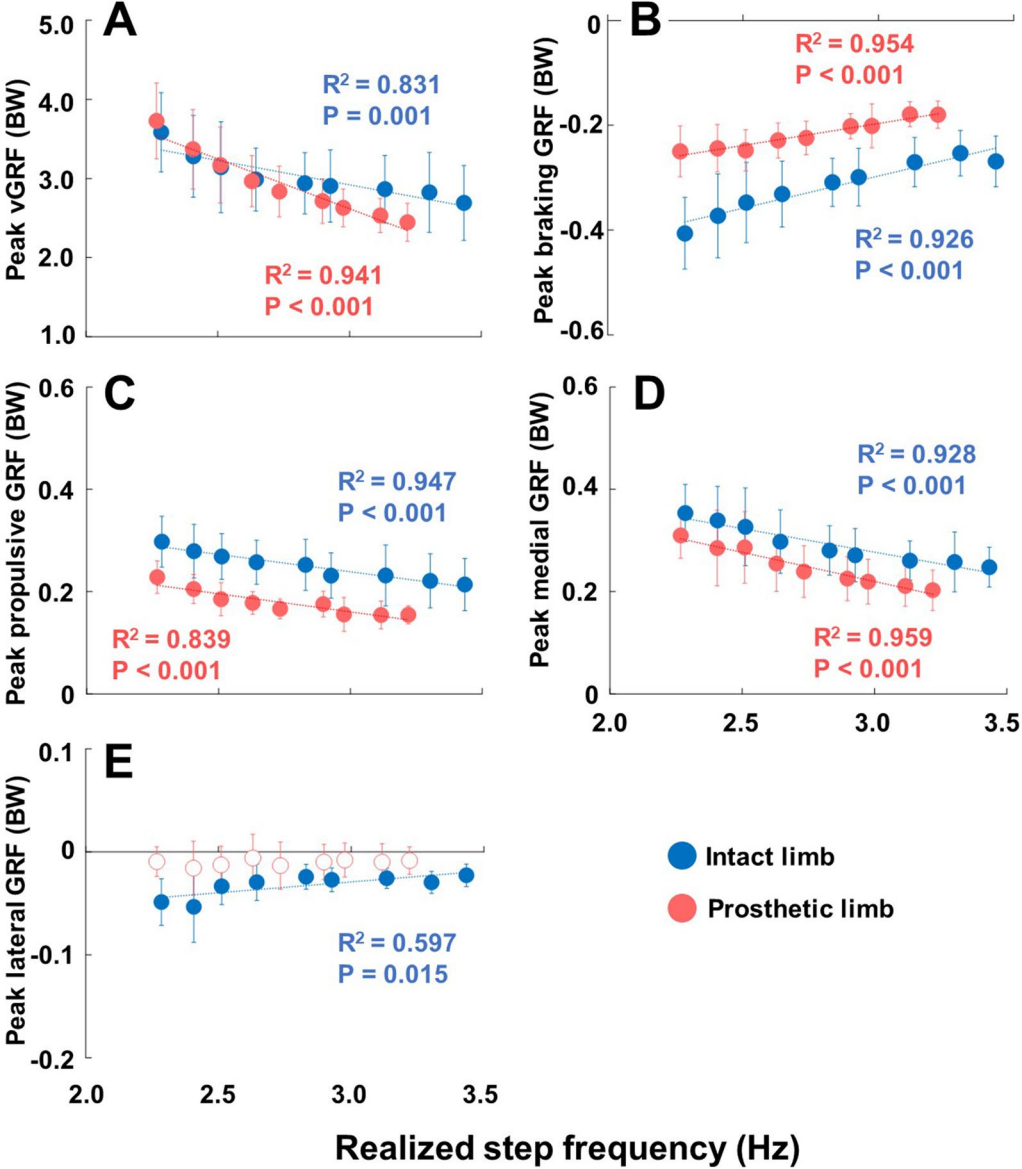
Mean peak ground reaction force parameters and linear regressions of both limbs across mean realized step frequencies at each metronome frequency. In every plot, the horizontal axis represents realized step frequency. Each plot represents a different parameter. Parameters are represented in respective vertical axis as: peak vertical GRF (BW) (**A**), peak braking GRF (BW) (**B**), peak propulsive GRF (BW) (**C**), peak medial GRF (BW) (**D**), and peak lateral GRF (BW) (**E**). Each circle indicates the mean of all the participant’s data at each increment of metronome frequency from − 20% to + 20%, with blue indicating intact limb, and red indicating prosthetic limb. Significant main effects are indicated by filled circles, while main effects that were not significant are indicated by unfilled circles. Relevant R^2^ and P values for the linear regressions are also displayed in matching color to the intact or prosthetic limb. P < 0.001 indicates P values which were at or smaller than 4 decimal places

Mean values and linear regressions with R^2^ values of each ground reaction force parameter across the mean realized step frequencies at each metronome frequency are presented in Fig. 5. All the ground reaction force parameters, except for the peak lateral GRF of the prosthetic limb (shown by the unfilled red circles in Fig. 5), demonstrated significant main effects, which are indicated by filled red (prosthetic limb) and blue (intact limb) circles. All parameters, except for the peak lateral GRF of the prosthetic limb, exhibited statistical significances with realized step frequency (P < 0.05) in linear regression analyses. The parameters: peak vertical GRF (vGRF) (A) (prosthetic limb R^2^ = 0.941, P < 0.001; intact limb R^2^ = 0.831, P < 0.001), peak propulsive GRF (C) (prosthetic limb R^2^ = 0.839, P < 0.001; intact limb R^2^ = 0.947, P < 0.001), and peak medial GRF (D) (prosthetic limb R^2^ = 0.959, P = 0.001; intact limb R^2^ = 0.928, P < 0.001), displayed a decreasing linear relationship with the increase in realized step frequency. For peak braking GRF (B) (prosthetic limb R^2^ = 0.954, P < 0.001; intact limb R^2^ = 0.926, P < 0.001) and peak lateral GRF (E) of the intact limb (R^2^ = 0.597, P = 0.015), the relationship had an increasing trend. For peak vGRF (A), the prosthetic limb exhibited higher vGRFs at realized step frequency of − 20% metronome frequency than the intact limb and lower vGRFs at realized step frequency of + 20% metronome frequency. The magnitude of peak braking GRF (B) tended to be smaller with increasing realized step frequency in both limbs. Also, the magnitude of difference in peak braking GRF between limbs was smaller at relatively higher step frequency and showed the clearest convergence towards symmetry among the GRF parameters. Both limbs exhibited almost linear trends. For both peak propulsive GRF (C) and peak medial GRF (D) parameters, the prosthetic limb had a lower magnitude of values than the intact limb across realized step frequencies, but both limbs appeared to be decreasing at a similar rate as realized step frequency increases. For the peak lateral GRF (E) parameter, only the intact limb demonstrated statistical significance with a positive trend towards zero. It should also be noted that for all significant parameters, the magnitude of GRFs decreased in relation to increasing realized step frequency. Correlation coefficients, mean (SD), median (IQR), and 95% confidence intervals (95% CI) of the GRF parameters are summarized in Additional file 3: Table S2.

### Ground reaction force impulse parameters

**Fig. 6:**
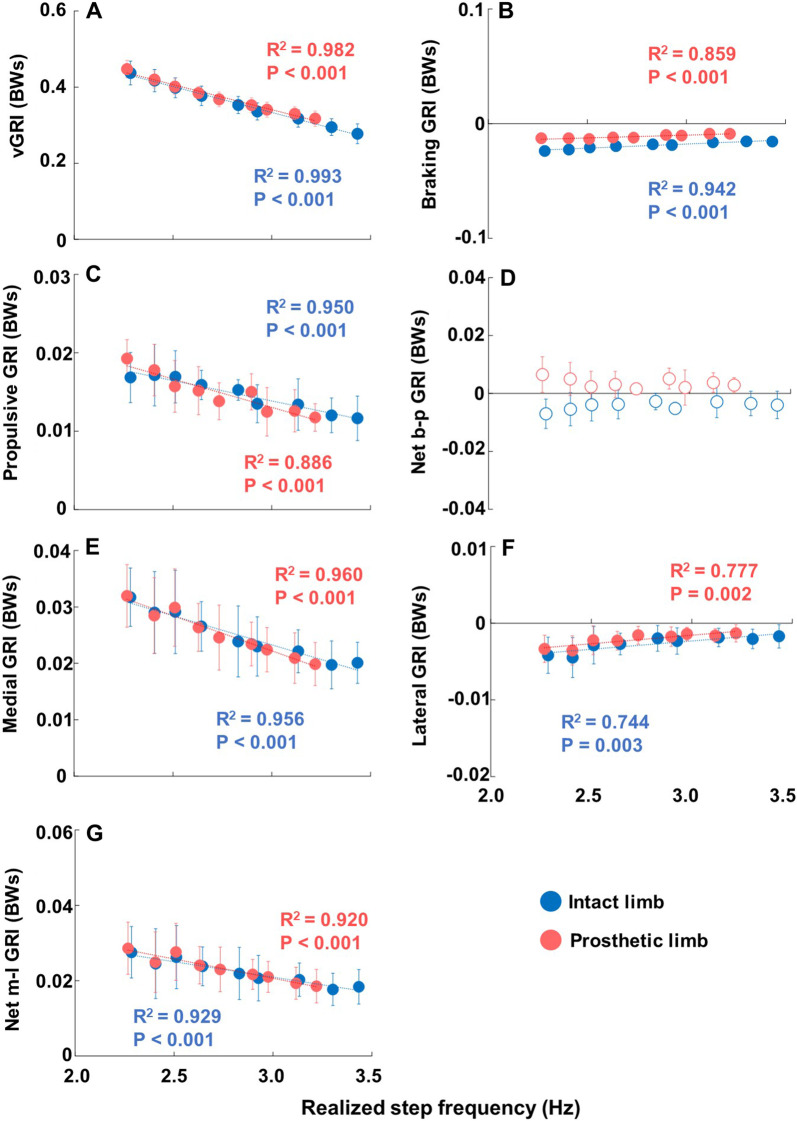
Mean ground reaction force impulse (GRI) parameters and linear regressions of both limbs across mean realized step frequencies at each metronome frequency. In every plot, the horizontal axis represents realized step frequency. Each plot represents a different parameter. Parameters are represented in respective vertical axis as: vertical GRI (BWs) (**A**), braking GRI (BWs) (**B**), propulsive GRI (BWs) (**C**), net braking-propulsive GRI (BWs) (**D**), medial GRI (BWs) (**E**), lateral GRI (BWs) (**F**), and net medial–lateral GRI (BWs) (**G**). Each circle indicates the mean of all the participant’s data at each increment of metronome frequency from − 20% to + 20%. Blue and red plots indicate intact and prosthetic limb, respectively. Significant main effects are indicated by filled circles, while main effects that were not significant are indicated by unfilled circles. Relevant R^2^ and P values for the linear regressions are also displayed in matching color to the intact or prosthetic limb. P < 0.001 indicates P values which were at or smaller than 4 decimal places

Mean values and linear regressions with R^2^ values of each ground reaction force impulse (GRI) parameter across the mean realized step frequencies at each metronome frequency are presented in Fig. 6. All the GRI parameters, except for the net b-p (braking–propulsive) GRI (D) of the prosthetic and intact limbs (shown by unfilled red and blue circles in Fig. 6), demonstrated statistical main effects, which are indicated by filled red (prosthetic limb) and blue (intact limb) circles. All parameters that demonstrated significant main effects exhibited statistical significances with realized step frequency (P < 0.05) in linear regression analyses. Except for braking GRI (B) and lateral GRI (F), all significant parameters had a negative linear association as realized step frequency increased. The braking GRI (B) (prosthetic limb R^2^ = 0.859, P < 0.001; intact limb R^2^ = 0.942, P < 0.001) and lateral GRI (F) (prosthetic limb R^2^ = 0.777, P = 0.002; intact limb R^2^ = 0.744, P = 0.003) had positive linear association towards zero with increasing realized step frequency. The magnitude of braking GRI (B) also is lower for the prosthetic limb at all increments of realized step frequency compared to the intact limb. For vertical GRI (vGRI) (A), the prosthetic limb (R^2^ = 0.982, P < 0.001) had a higher magnitude when compared to the intact limb (R^2^ = 0.993, P < 0.001) as realized step frequency increased. The values of the propulsive GRI (C) over increasing realized step frequency did not show remarkable differences between the prosthetic limb and the intact limb. The prosthetic limb (R^2^ = 0.886, P < 0.001) showed a higher value at realized step frequency of -20% metronome frequency, and intact limb (R^2^ = 0.950, P < 0.001) had close values to the prosthetic limb at realized step frequency of + 20% metronome frequency. The values of the prosthetic and intact limbs for medial GRI (E) were similar. The linear association of the prosthetic limb (R^2^ = 0.960, P < 0.001) and the intact limb (R^2^ = 0.956, P < 0.001) were also similar. For the lateral GRI (F), both limbs had a positive association with realized step frequency with the prosthetic limb (R^2^=0.777, P=0.002) showing close value to the intact limb (R^2^=0.744, P=0.003) as realized step frequency increased. The net m-l (medial–lateral) GRI (G) values for the prosthetic limb (R^2^ = 0.920, P < 0.001) are similar to the intact limb (R^2^ = 0.929, P < 0.001) at all increments of realized step frequency and both linear models decreased at a similar rate. Correlation coefficients, mean (SD), median (IQR), and 95% confidence intervals (95% CI) of the GRl parameters are summarized in Additional file 4: Table S3.

### Asymmetry ratio of the parameters

**Fig. 7:**
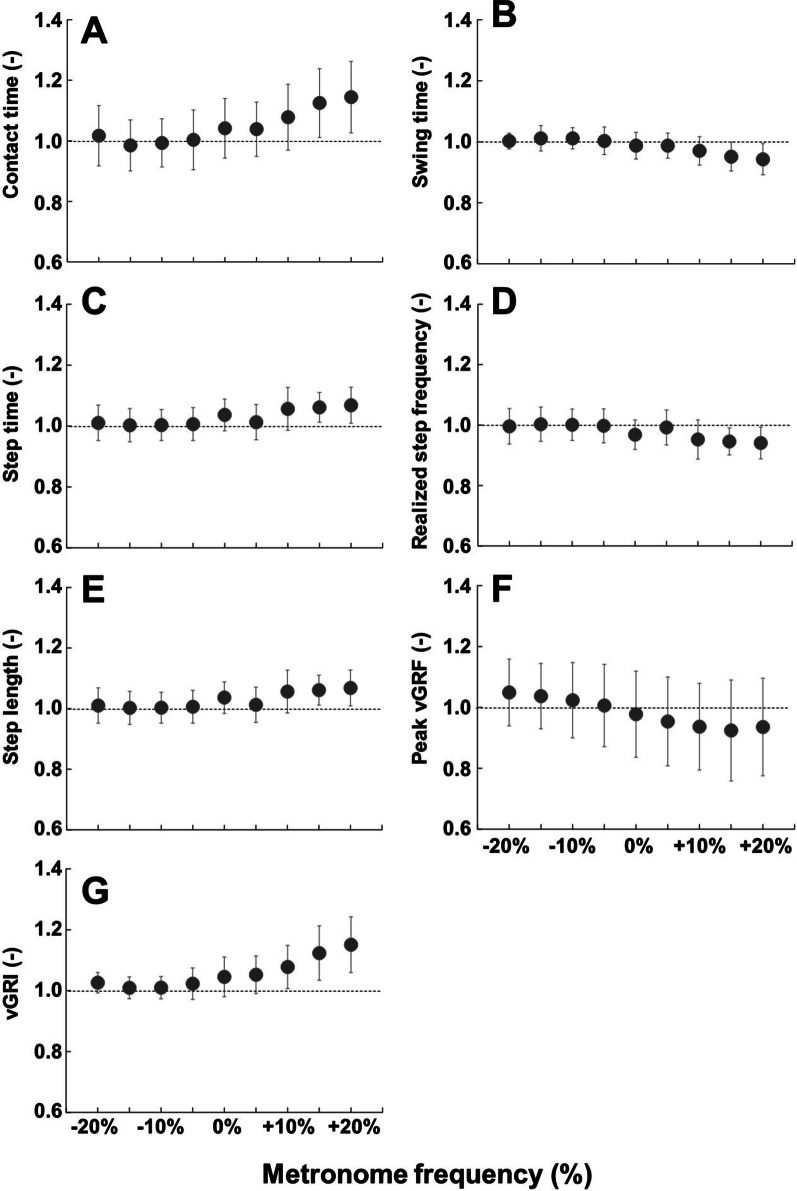
Asymmetry ratio of parameters that demonstrated significant main effects. Plot **A** is contact time, plot **B** is swing time, plot **C** is step time, plot **D** is step frequency, plot **E** is step length, plot **F** is peak vertical GRF, and plot **G** is vertical GRI. In every plot, the horizontal axis represents metronome frequency from − 20 to + 20%, with the vertical axis representing the ratio between limbs by dividing the mean of prosthetic limb data by the mean of intact limb data for each parameter, resulting in 1.0 representing perfect symmetry. Note that + means more toward the prosthetic side and vice versa

The parameters that demonstrated statistical main effects in the asymmetry ratio are presented in Fig. 7. Due to the calculation of this parameter given in the methods section, 1.0 denotes perfect symmetry. The asymmetry ratio of seven parameters demonstrated significant main effects, which are indicated by filled circles in Fig. 7. Except for swing time (B), step frequency (D), and peak vGRF (F), other parameters appeared to become prosthetic limb dominant at higher metronome frequencies. For swing time (B) and step frequency (D) the intact limb became more dominant as metronome frequency increased. Peak vGRF (F) favored the prosthetic limb at − 20% metronome frequency, but favored the intact limb at + 20% metronome frequency, with the most symmetric value between -5% to 0% metronome frequency. Although swing time (B), step time (C), step frequency (D), and step length (E) seemed to have relatively symmetric values at − 20% to − 5% metronome frequency, contact time (A) and vGRI (G) appeared to have the most symmetric values occurring in metronome frequency between − 10% and − 5%. Correlation coefficients, mean (SD), median (IQR), and 95% confidence intervals (95% CI) of the asymmetry ratio of the parameters are summarized in Additional file 5: Table S4.

## Discussion

The first aim of this study was to investigate the effects of step frequency on the magnitude of GRFs in the intact and prosthetic limbs of individuals with transfemoral amputation. Our results showed that increasing step frequency had significant main effects in the reduction of the magnitude of GRFs in both limbs, except peak lateral GRF in the prosthetic limb (Fig. 5). This was in line with our first hypothesis that increasing step frequency at a given speed would lower the magnitude of GRFs on the intact and prosthetic limbs. This relationship could be explained by natural bouncing gait during running [48]. As step frequency increases during constant velocity, the stiffness of the leg increases, resulting in the reduction of the vertical displacement of the center of mass [35]. The lowered displacement of the center of mass reduces the GRFs the body undertakes upon contact with the ground. Our results are also consistent with previous studies that investigated the effects of increased step frequency on gait parameters in able-bodied individuals [20–24]. Therefore, current results indicate that increasing step frequency has similar effects on GRFs in both individuals with and without amputation.

The second aim of this study was to investigate the effect of step frequency on the symmetry of GRFs and spatiotemporal parameters between intact and prosthetic limbs. As shown in Fig. 7F, the peak vertical GRF asymmetry ratio was closest to 1.0 between − 5% and 0% of preferred step frequency. In addition, some parameters, such as contact time (Fig. 7A) and vGRI (Fig. 7G) showed minimized asymmetry between − 10% and − 5% of the preferred step frequency. These results partially support our second hypothesis that the asymmetry of GRFs between the intact and prosthetic limbs would be minimized at the preferred step frequency. Previous studies that found asymmetric gait being more pronounced in individuals with amputation [9, 10] mainly focused on differences between the intact and prosthetic limb, but did not consider the effects of step frequency. Due to the nature of bouncing gait, the vertical displacement of the center of mass is affected by the stiffness of the leg [35]. However, the prosthetic limb’s stiffness cannot be modified due to the absence of biological ankle and knee joints. This could cause the additional asymmetry as there is a need for the intact limb to compensate for additional forces generated by the unadaptable stiffness of the prosthetic limb. Future studies on asymmetric gait in individuals with amputation should consider step frequency, as our study has found that this also has an impact on asymmetric gait. However, the mechanism of how step frequency affects each gait parameter is not clear and further investigation is warranted.

A metronome was used to control the step frequency in this study. The realized step frequency was assumed to be constant between the prosthetic and intact limb, but our results showed otherwise. Realized step frequency of the prosthetic and intact limbs did not increase at a similar rate over increasing metronome frequency. The error analyses of realized step frequency showed that both intact and prosthetic limbs generally had more percent errors at higher or lower metronome frequencies (Fig. 2). This means that the realized step frequency of both limbs did not increase in exact 5% increments. Therefore, the lack of adaptation to each step frequency may have affected the results of our study, and this is one of the limitations of our study [30].

Regarding the spatiotemporal parameters, contact time (Fig. 4A) tended to be constant across realized step frequencies, but aerial time (Fig. 4B) decreased constantly as realized step frequency increased in both limbs. This suggests that the individuals with amputation coped with increased step frequency by reducing swing time (Fig. 4C), but maintained contact time for balance and stability. As swing time decreased over increasing realized step frequency, step time and step length also decreased because running speed was kept constant.

For ground reaction force impulses (GRIs), all statistically significant parameters displayed a trend of change towards zero as realized step frequency increased (Fig. 6). One explanation is that the ground reaction forces decreased as realized step frequency increased. This may be due to the lowered magnitude of impulse per step for acceleration required to maintain speed, as the total amount of impulse can be spread among the larger number of steps taken (Fig. 6C). Our results for running propulsive GRI seem to disagree with prior studies on walking that intact leg impulses to be higher than prosthetic leg impulses [18, 43, 49]. This may have been affected by the relationship between leg stiffness and step frequency as discussed earlier. A previous study suggested that increased GRI on the intact side is a larger factor for injury and degenerative disease in individuals with transfemoral amputation, while increased peak GRF is a larger factor in individuals with transtibial amputation [50]. This leads us to the clinical applications for the reductions of GRFs or GRIs in individuals with amputation. Knee osteoarthritis is much more prevalent in the population of individuals with amputation [51, 52], and studies have shown higher GRFs [53] or GRIs [50] to be a possible cause. A study that was done in able-bodied individuals also found correlations between higher GRFs and stress fractures [22]. Although there may be a variety of factors such as prosthetic components [54, 55], physical conditions [56, 57], and other gait parameters such as velocity and motor control [54, 58] that can affect GRFs and GRIs on individuals with amputation, our results indicate that increasing step frequency is a viable strategy to reduce GRFs or GRIs. Therefore, reduction of GRFs or GRIs by increasing step frequency may have implications to reduce potential injury risk during running in individuals with amputation. Future studies should investigate the optimal relationship between running speed and step frequency to minimize injury risk through comprehensive kinetic and kinematic analyses.

The instrumented treadmill was used to control the running speed while varying step frequency. Therefore, we are not able to generalize our study's data to overground running. While there are few studies on the generalization of treadmill running to overground running in amputees, studies on able-bodied individuals found no statistical differences between the majority of kinetic and kinematic parameters in the two modalities of running conditions [59–61]. In our study, we allowed five minutes or more for the individuals with amputation to habituate to treadmill running, according to prior studies [62–64]. While some studies found differences in the use of instrumented treadmills between individuals with and without amputation such as variability in gait asymmetry and increased energy costs [59, 65, 66], measurements using instrumented treadmills appeared to be valid for gait analyses [59, 67].

Several other limitations should be taken into account when interpreting our results. We had nine participants (Table 1) in our study which limited the statistical power, but this is common for studies on running in individuals with amputation. Previous studies on running in individuals with amputation had similar small sample sizes due to the difficulty of recruiting participants that meet study criteria [13, 20, 26, 29]. The participants with unilateral transfemoral amputation recruited for the study were also experienced members of track and field teams. However, some recruits were more experienced compared to others. Care should be taken before applying our results to the general population of individuals with amputation.

## Conclusions

It is found that increasing step frequency (from − 20% to + 20% of preferred step frequency) on individuals with amputation decreases the magnitude of GRFs, similar to prior studies on able-bodied individuals. Further, minimized gait asymmetry can be observed in peak vertical GRF between − 5% and 0% of the preferred step frequency, and in contact time and vertical ground reaction impulse between − 10% and − 5% of the preferred step frequency during running. However, individuals with amputation were not able to match the frequency of the intact and prosthetic limbs at higher step frequencies. Therefore, while increased step frequency can reduce GRFs overall, it introduces further asymmetry in individuals with amputation. Further study is needed to investigate the optimal step frequency for the prevention of injury while running.

## Supplementary Information

**Additional file 1: Figure S1.** Schematic definition of spatiotemporal parameters. A: Illustration of a sprinter with unilateral transfemoral amputation during the contact and aerial phases for the intact (blue) and prosthetic (red) limbs. Tcontact, Taerial, Tstep and Tswing indicate contact, aerial, step and swing time, respectively. B, Corresponding vGRF data for the intact and prosthetic limbs in a representative two steps while running at preferred step frequency (in 2.11 m/s) recorded from one participant. The vGRF data were normalized to the BW.

**Additional file 2: Table S1.** Descriptive statistics for spatiotemporal parameters. Correlation coefficient, mean (SD), median (IQR), and 95% confidence intervals (95%CI) in both limbs at all metronome frequencies are presented. Since calculation of 95%CI are based on normal distribution, we made several cells blank at a particular frequency condition, where the data were not normally-distributed. * p< 0.05, ** p< 0.01.

**Additional file 3: Table S2.** Descriptive statistics for Peak GRF. Correlation coefficient, mean (SD), median (IQR), and 95% confidence intervals (95%CI) in both libs at all metronome frequencies are presented. Since calculation of 95%CI are based on normal distribution, we made several cells blank at a particular frequency condition, where the data were not normally-distributed. *p< 0.05, **p< 0.01.

**Additional file 4: Table S3.** Descriptive statistics for GRI. Correlation coefficient, mean (SD), median (IQR), and 95% confidence intervals (95%CI) in both limbs at all metronome frequencies are presented. Since calculation of 95%CI are based on normal distribution, we made several cells blank at a particular frequency condition, where the data were not normally-distributed. **p< 0.01.

**Additional file 5: Table S4.** Descriptive statistics for Asymmetry ratio. Correlation coefficient, mean (SD), median (IQR), and 95% confidence intervals (95%CI) in both limbs at all metronome frequencies are presented. Since calculation of 95%CI are based on normal distribution, we made several cells blank at a particular frequency condition, where the data were not normally-distributed. **p < 0.01.

**Additional file 6.** Equations for temporal parameters.

## Data Availability

The datasets used and/or analyzed during the current study are available from the corresponding author on reasonable request.

## Abbreviations

BW: Body weight
*f*_step_: Step frequency
GRF: Ground reaction force
GRI: Grund reaction force impulse
Net b-p GRI: Net braking-propulsive ground reaction force impulse
Net m-l GRI: Net medial–lateral ground reaction force impulse
RSP: Running specific prosthesis
vGRF: Vertical ground reaction force
vGRI: Vertical ground reaction force impulse
