# Supplementary figures and images for: Effects of step frequency during running on the magnitude and symmetry of ground reaction forces in individuals with a transfemoral amputation
Source: J Neuroeng Rehabil. 2022 Mar 23;19:33. doi: 10.1186/s12984-022-01012-8 (PMC8944140; doi:10.1186/s12984-022-01012-8)

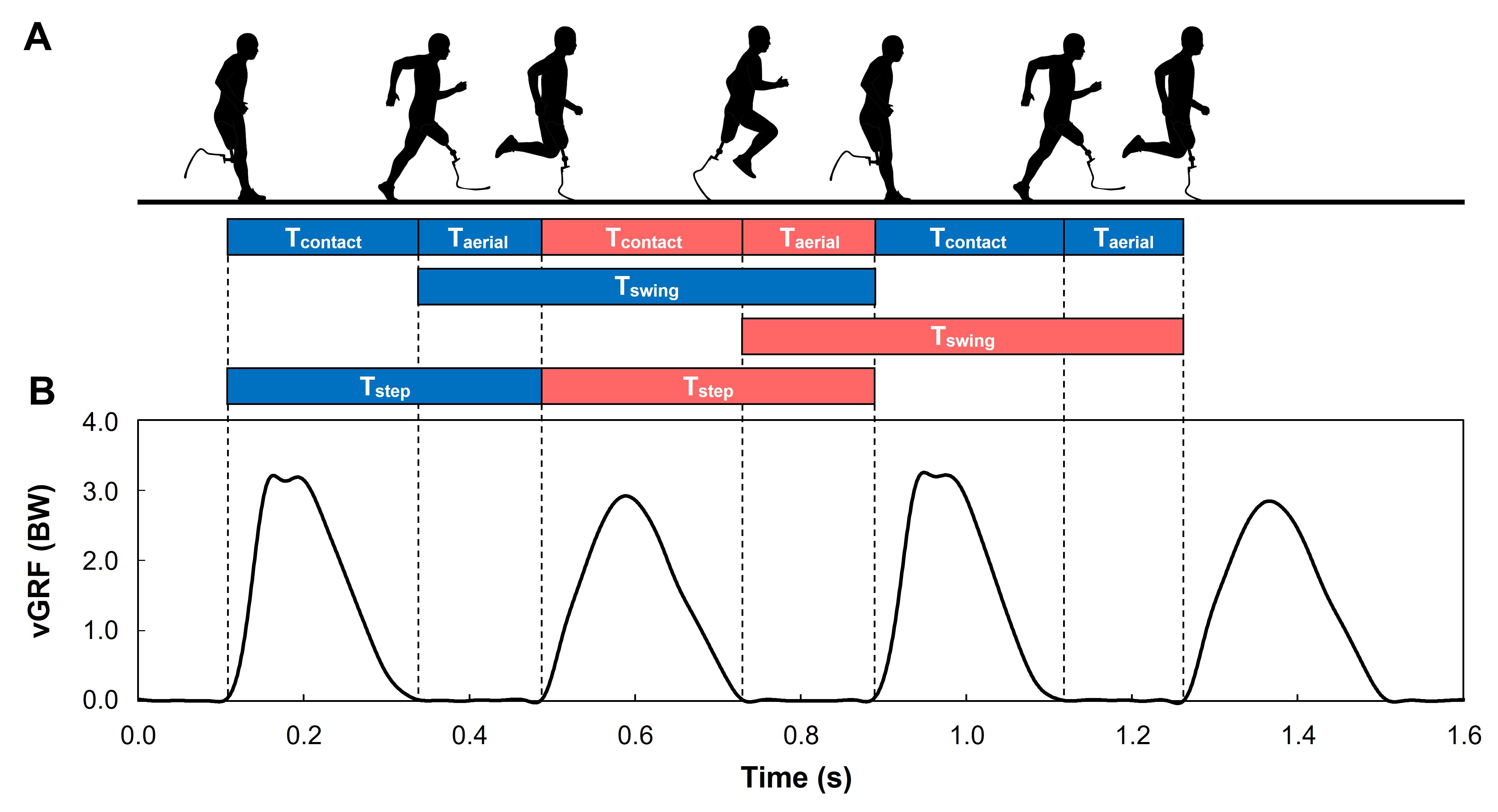

Supplement: Supplementary file 1 — Additional file 1: Figure S1. Schematic definition of spatiotemporal parameters. A: Illustration of a sprinter with unilateral transfemoral amputation during the contact and aerial phases for the intact (blue) and prosthetic (red) limbs. Tcontact, Taerial, Tstep and Tswing indicate contact, aerial, step and swing time, respectively. B, Corresponding vGRF data for the intact and prosthetic limbs in a representative two steps while running at preferred step frequency (in 2.11 m/s) recorded from one participant. The vGRF data were normalized to the BW. [file 12984_2022_1012_MOESM1_ESM.jpg]
