# Supplementary material for: Effects of step frequency during running on the magnitude and symmetry of ground reaction forces in individuals with a transfemoral amputation
Source: J Neuroeng Rehabil. 2022 Mar 23;19:33. doi: 10.1186/s12984-022-01012-8 (PMC8944140; doi:10.1186/s12984-022-01012-8)
Supplement: Supplementary file 6 — Additional file 6. Equations for temporal parameters. [file 12984_2022_1012_MOESM6_ESM.docx]

**Supplementary materials: Equations for temporal parameters**

In this study, temporal parameters for both intact (_INT) and prosthetic (_PST) limbs were calculated using following definitions and equations. First, we determined contact time (T_contact_) as the time that the applied force exceeded the threshold on the force platforms and aerial time (T_aerial_) as the time interval between the end of the contact period of one foot and the beginning of the contact period of the opposite foot (Figure S1). Next, we also determined swing time (T_swing_) for both limbs as the time interval between foot-off and ipsilateral foot contact as:

T_swing__INT = T_aerial__INT + T_contact__PST + T_aerial__PST (1)

T_swing__PST = T_aerial__PST + T_contact__INT + T_aerial__INT (2)

Finally, we calculated step time (T_step_) as the time interval between successive heel contacts and realized step frequency as the inverse of the time from touchdown to contralateral touchdown, which was equal to the sum of the contact time (T_contact_) and subsequent aerial time (T_aerial_):

T_step_ = T_contact_ + T_aerial_ (3)

Freq_step_ = 1 / (T_contact_ + T_aerial_) (4).
